# Silencing of Her2, CCNB1 and PKC Genes by siRNA Results in Prolonged Retardation of Neuroblastoma Cell Division

**Published:** 2011

**Authors:** I.A. Akimov, E.L. Chernolovskaya, Yu.E. Spitsyna, E.I. Ryabchikova, M.A. Zenkova

**Affiliations:** Institute of Chemical Biology and Fundamental Medicine, Siberian Branch, Russian Academy of Sciences

**Keywords:** neuroblastoma, siRNA, *Her2*, *CCNB1*, *PKC*, proliferation

## Abstract

Deregulation of the expression of the genes that are involved in the control of the cell cycle impairs cellular differentiation and leads to cell death. This process can result in uncontrollable cell proliferation and, subsequently, cancer development. In this study, we examined the effect of the silencing of cancer-related genes by small interfering RNAs (siRNA) targeted at mRNA**of* Her2*, cyclin B1 (*CCNB1*), and protein kinase C*(PKC) *on the proliferation of human cancer cells of different origins. Maximum silencing of*CCNB1*,*Her2*(in KB-3-1, SK-N-MC, MCF-7 cells), and*PKC*(in MCF-7 cells) was achieved 72 h after transfection of the corresponding siRNAs, and 12 days after the transfection, the initial levels of the target mRNAs were fully recovered. Silencing of*Her2*,*CCNB1,*and*PKC*differently effected the proliferation of the cell lines under study. The most pronounced antiproliferative action of the investigated siRNAs was observed in neuroblastoma SK-N-MC cells (3 – 10-fold reduction in the proliferation rate) even after the recovery of the initial levels of expression of**the* Her2*,*CCNB1, *and*PKС *genes. The obtained data indicate that the*CCNB1 *and*PKC*genes can be used as targets in the development of drugs for neuroblastoma treatment.

## INTRODUCTION

Malignant cell transformation is a complex process involving both genetic disorders and failure in the regulation of differentiation, apoptosis, and proliferation [1, 2]. The regulatory signal transduction network in a cell is cascade-like and consists of a number of duplicating paths [3]. When silencing one of the cell factors that participate in signal transduction, its function can be compensated by the activation of alternative signal paths [3]. While on one hand this further complicates the search for adequate molecular targets , on the other hand, it makes it necessary to design anti-tumor cells that would provide an irreversible antiproliferative effect by “switching off” the synthesis of the protein factors localized at the points of interception of the regulatory paths.

Hyperexpression of normal genes or expression of their mutant variants encoding transcription factors, receptors, tyrosine kinases, and other regulatory proteins can be behind the uncontrollable cell division upon cancer [2]. Suppression of the synthesis of these proteins may provide a positive effect and normalize cell proliferation [4–7].

Today, RNA interference is widely used both to study the role of genes in the regulation of the cell cycle and to reveal potential targets for designing new therapeutical agents [8–10]. Specific and efficient silencing of target genes can be achieved using chemically synthesized small interfering RNAs (siRNAs) [11]. The products of such genes as *Her2, * cyclin B1( *CCNB1* ), and protein kinase C ( *PKC* ) belong to different groups of proteins that participate in the regulation of the cell cycle [8, 12, 13]. Earlier, it was experimentally demonstrated and clinically verified that disorders in the expression of these genes may result in the emergence of malignant tumors in humans [12, 14–18]. The level of amplification and expression of these genes in breast or ovarian cancer cells, or cancer cells in other human organs, is considerably higher than that in the normal cells of these organs [14, 17–32]. Moreover, high levels of expression of the *Her2* , *CCNB1* , and *PKC * genes correlate with dire prognosis: three-year survival rate, and the recurrence-free period shortens [17, 18, 22–25, 30].

The * Her2 * gene (also known as *c-erb-B2 * and *neu* ) encodes a transmembrane glycoprotein possessing tyrosine kinase activity and belonging to the family of human epidermal growth factor receptors, which play a significant role in the regulation of the proliferation, differentiation, and mobility of human epitelial cells [33, 34]. A level of *Her2 * gene expression considerably higher than the normal level was detected in the cells of humans with breast, endometrial, uterine neck, ovarian, fallopian tube, and lung cancer [25, 29, 30].

Cyclin B1 encoded by the *CCNB1 * gene is a regulatory subunit of the cyclin-dependent kinase complex (CDK1) that regulates the transition from phase G _2_ of the cell cycle into phase M [35]. Hyperexpression of the *CCNB1 * gene usually does not immediately result in cell cycle disorder, which causes the accumulation of mutations in a cell [36]. An increased expression level of this gene, which is typical of benign and malignant human prostate tumors [37], is often the reason for aneuploidy [38]. Disturbance in the *CCNB1 * geneexpression could be regarded as an early warning in malignant cell transformation [17].

Protein kinase C encoded by the *PKC* gene is expressed in many human tissues and organs; it plays an important role in the transduction of the regulatory signals that activate various cell functions, including proliferation [39, 40]. A level of *PKC* gene expression higher than the normal level was detected in human cancer cells of different origins [18, 19].

It has been known that silencing of the same gene in tumor cells of different tissue origins may result in various antiproliferative effects [41]. This determines the necessity for comparing the antiproliferative action of siRNAs in different human tumor cell lines. It was earlier demonstrated that siRNAs targeted at mRNAs of the *Her2* , *CCNB1* , and *PKC* genes efficiently silence the target genes and have an antiproliferative effect on human cancer cells for 5 days following transfection [41].

In this study, we assessed the long-term consequences of short-term silencing of *Her2* , *CCNB1* , and *PKC * on the proliferation and morphology of human tumor cells. We demonstrated that siRNAs that are homologous to mRNAs of the *Her2* , *CCNB1* , and *PKC* genes silence these genes, attaining maximum effect (up to 4–22% of the control level) 72 h after transfection. These siRNAs demonstrated different efficiencies of deceleration of the division of human tumor cells of different tissue origins. We found that the antiproliferative effect of siCyc and siPKC in SK-N-MC neuroblastoma cells is retained even after the initial levels of target gene expression have been recovered. The data obtained permit the reasonable assumption that the *CCNB1 * and * PKC* genes play a key role in sustaining a high proliferation rate of neuroblastoma cells, whereas their short-term silencing results in change in signal transmission paths and normalization of the rate of cell division. Thus, the *CCNB1* and * PKC * genes in SK-N-MC cells can serve as potential efficient targets for the agents targeted at neuroblastoma, including siRNAs.

## EXPERIMENTAL

**siRNAs**

All the oligonucleotides that were used to form siRNA duplexes were synthesized at the Laboratory of RNA Chemistry, Institute of Chemical Biology and Fundamental Medicine, Russian Academy of Sciences, Siberian Branch using the solid-phase phosphiteamide method on an automatic synthesizer ASM-102U (Biosset, Russia) and extracted using high-efficiency reversed phase chromatography. The nuclease-sensitive sites in siRNA were protected by introducing 2’-O-Ме-analogues of ribonucleotides into the siRNAs using the algorithm described earlier [42, 43]. According to the data of electrophoresis in polyacrylamide gel under denaturating conditions, the purity of the oligoribonucleotides was at least 95%. The following siRNAs were used in the present study: siHer homologous to the region 1297–1317 of mRNA of the human *Her2 * gene (sense strand 5’-GCAGUUACCAGUGCCAAUAUU-3’, antisense strand 5’-UAUUGGCACUGGUAACUGCCC-3’); siCyc homologous to the region 189–209 of mRNA of the human *CCNB1* gene (sense strand 5’-CACCAGGAACUCGAAAAUUUU-3’, antisense strand 5’-AAUUUUCGAGUUCCUGGUGAC-3’); and siPKC homologous to region 1079–1099 of mRNA of the human * PKC * gene (sense strand GCGGCCAGAGAAGGAAAAAUU-3’, antisense strand 5’-UUUUUCCUUCUCUGGCCGCUG-3’), 2’-O-Me-modified units are underlined. siScr (sense strand 5’-CAAGUCUCGUAUGUAGUGGUU-3’, antisense strand 5’-CCACUACAUACGAGACUUGUU-3’) without significant homologies with the nucleotide sequences of mRNAs of mouse, rat, and human genes was used as the negative control. siRNAs were selected using the BioPredSi software [44]. siRNAs were obtained via fusion of the antisense and sense strands in a buffer of 15 mM HEPES-KOH pH 7.4, 50mM potassium acetate, and 1 mM magnesium acetate. To perform this procedure, equimolar mixtures of oligoribonucleotides (sense and antisense strands) were incubated for 2 min at 90 ^0^ С and slowly cooled to room temperature.

**Cell cultures and siRNA transfection**

Cell lines of human uterine neck carcinoma KB-3-1, SK-N-MC neuroblastoma, and MCF-7 breast adenocarcinoma were obtained from the collection of the Institute of Cytology of the Russian Academy of Sciences (St. Petersburg, Russia). The cells were cultivated in a DMEM medium (Dulbecco’s Modified Eagle Medium) containing 10% of fetal bovine serum (FBS), 100 u/ml of penicillin, 100 µg/ml of streptomycin, and 0.25 µg/ml of amphotericin at 37 ^о^ C in humid atmosphere with 5% CO _2 _ content. Twenty-four hours prior to the experiment, the cells in the phase of exponential growth were seeded in 6-well plates: KB-3-1 – 4 × 10 ^4^ , SK-N-MC – 2 × 10 ^5^ , MCF-7 – 8 × 10 ^4 ^ cells/well or in 24-well plates: KB-3-1 –10 ^5^ , SK-N-MC – 1.25 × 10 ^5^ , and MCF-7 – 1.5 × 10 ^5 ^ cells/well and allowed to adhere overnight. The cells were transfected with siRNA at a concentration of 200 nM; Lipofectamine 2000 ^ТМ^ (Invitrоgen, United States) or Oligofectamine ^ТМ^ (Invitrоgen, United States) for SK-N-MC cells were used as transfection agents, in accordance with the manufacturer’s protocol. The levels of specific mRNAs were determined 1–5, 7, 10, and 12 days after transfection. The cells treated only with a transfection agent or siScr/lipofectamine (oligofectamine) complex were used as the control. During the experiment, the cells were reseeded once in 3–4 days to maintain the exponential growth.

**Real-time reverse transcription PCR (RT-PCR)**

The total RNA was extracted from the cells using the SDS-phenol method [45]. Reverse transcription (RT) reaction was carried out in a 20 µl mixture containing 1 µg of total RNA, 5 µM of the oligo(dT _15_ ) primer, 50 mM of Tris-HCl, pH 8.3, 75 mM of KCl, 3 mM of MgCl _2_ , 0.5 mM of dNTP, 5 mM of dithiothreitol, and 10 U of M-MLV reverse transcriptase from the Moloney murine leukemia virus. The reaction mixture was incubated at 42 ^ о^ C for 1 h. The resulting cDNA was amplified in a reaction mixture (volume 20 µl) containing 1 µl of cDNA, 10 mM of Tris-HCl, pH 8.3, 50 mM of KCl, 1.5 mM of MgCl _2_ , 0.01% Tween-20, 0.25 mM of each dNTP, 0.25 µM of each primer, 0.5 mM of EvaGreen (Biotium, United States), and 2 U of thermostable DNA polymerase *Thermus aquaticus * (produced at the Institute of Chemical Biology and Fundamental Medicine, Siberian Branch, Russian Academy of Sciences). Real-time PCR was carried out on a Bio-Rad iQ5 Multicolor Real-Time PCR Detection System instrument according to the following scheme: one cycle – 3 min, 95 ^о^ C, 40 cycles – 30 s, 95 ^о^ C, 30 s – 58 ^о^ C, 30 с – 72 ^о^ C. The amount of mRNA of each gene was standardized per the amount of mRNA of β-actin, since the level of expression of this gene is relatively constant for different types of cells. The relative level of gene expression was determined using the Bio-Rad iQ5 2.0 software (Bio-Rad Laboratories Inc., United States).

The following DNA primers were used in the present study:

*Her2* forward – 5’-AGCAATGGTGTCAGTATCCAGGCT-3’, 

*Her2* reverse – 5’-TGCAAATGGACAAAGTGGGTGTGG-3’, 

*CCNB1* forward – 5’-AGGAAGAGCAAGCAGTCAGACCAA-3’, 

*CCNB1 *reverse – 5’-GCAGCATCTTCTTGGGCACACAAT-3’, 

*PKC* forward – 5’-GCTGTCTTTCACGATGCCCC-3’, 

*PKC* reverse – 5’-CACCCGACGACCCTGAGAGA-3’, 

β-actin forward– 5’-ACCAACTGGGACGACATGGAGAAA-3’, 

β- actin reverse – 5’-TTAATGTCACGCACGATTTCCCGC-3’.

**MTT test**

The number of living cells was determined using the colorimetric method based on oxidation of 3-[4,5-dimethylthiazol-2-yl]-2,5-diphenyl tetrazolium bromide (MTT) in mitochondria of living cells [46]. On day 6 after siRNA transfection, the cells were seeded from the 6-well plate to a 96-well plate, with density being 1.5 × 10 ^3 ^ (KB-3-1), 7.5 × 10 ^3^ (SK-N-MC), and 3 × 10 ^3 ^ cells/well (MCF-7), followed by incubation over a period varying from 1 to 6 days at 37 ^о^ C. Then, the MTT solution was added to the cells until the concentration reached 0.5 mg/ml. After 3 h, the culture medium was removed; the resulting formazan crystals were dissolved in dimethyl sulfoxide (100 µl/well), and the optical density of the solution was measured on a Multiscan RC multichannel photometer (Labsystems) at wavelengths of 570 and 630 nm. The results were represented as the relative proliferation rate, i.e., the rate of cell division in the sample standardized to the cell division rate in the control (taken as 100%). The proliferation rate was calculated using the following formula: *V* = ( *D*_12 _ – *D*_7_ )/(Δ *t* ), where *D*_12_ and  *D*_7 _ are the optical densities in the wells 12 and 7 days after transfection, respectively; Δ *t * is the time interval of cell observation (i.e., 12–7 = 5 days).

**Microscopic analysis**

For the microscopic analysis, SK-N-MC neuroblastoma cells after transfection with siRNA preparations for 48 h were seeded (10 ^5 ^ cells/well)in round coverslips with a diameter of 15 mm, which were placed into the wells of a 24-well plate. The oligofectamine-only treated cells, intact cells, and the cells transfected with siScr (controls) were incubated for 24, 48, and 72 h; whereas the cells transfected with siRNA siCyc, siPKC were incubated for 3, 5, 7, and 12 days. After the incubation, the cells were washed with 0.5 ml of DMEM and immobilized without taking them off the coverslip with 4% paraformaldehyde in a DMEM medium. Cell preparations were then washed with PBS, treated with acetone for 5 min, and washed with PBS, again. Then, hematoxylin or Feulgen staining [47] was carried out, and the cells were incorporated into polystyrene. The stained preparations of SK-N-MC neuroblastoma cells were studied in a DM2500 light microscope with a DFC420 digital camera (Leica, Germany). Mitosis calculation was performed upon zooming ×40.

## RESULTS

**Fig. 1:**
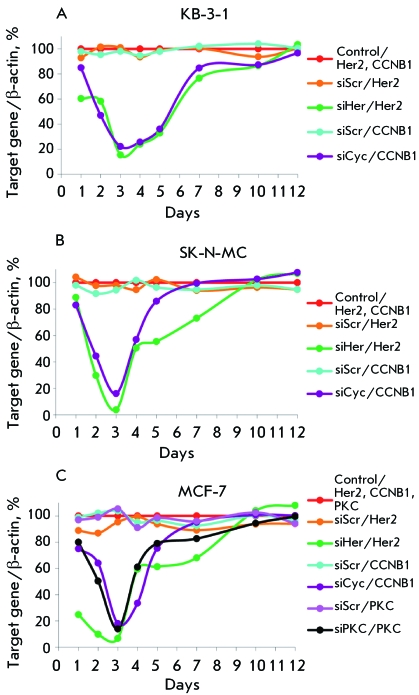
Relative levels of *Her2* , *CCNB1* and *PKC* mRNAs in KB-3-1 (A), SK-N-MC (B), and MCF-7 (C) cells 1 – 12 days after siRNAs (200 nM) transfection. The level of *β-actin* mRNA was used as an internal standard. Mean values obtained from three independent experiments. The standard error of the mean < 10%. Control/ *gene(s)* is the relative mRNA level of *genes* in the control cells. siRNA/ *gene(s)* is the relative mRNA level of *gene(s)* in the cells after siRNA transfection.

The *Her2* , * CCNB1, * and * PKC* genes encoding the most significant regulatory proteins of the cell cycle were selected as targets for siRNAs, since their hyperexpression is frequently associated with the emergence of various tumor diseases.

**Analysis of the expression levels of the**

*Her2* **,** *CCNB1,* **and** *PKC* **genes in KB-3-1, SK-N-MC, and MCF-7 cell lines after transfection with corresponding siRNAs**

The expression of target genes was determined in the following cell lines: KB-3-1 (uterine neck carcinoma), SK-N-MC (neuroblastoma), and MCF-7 (breast adenocarcinoma). We had previously detected a high mRNA level of the *CCNB1* gene in these cell lines and a slightly lower mRNA level of the *Her2 * gene; an increased mRNA level of the *PKC* gene was revealed only in MCF-7 cells [41].

The effect of siRNAs on the expression of target genes was analyzed on the basis of the following scheme: siRNA at a concentration of 200 nm was transfected to the cells; Oligofectamin ^ТМ^ (SK-N-MC) and Lipofectamin 2000 ^ТМ ^ (other cell lines) being used as transfection agents. 1–12 days after transfection (upon long-term experiments, the control cells were reseeded once per 3–4 days), real-time PCR was used to extract the total RNA from the cells and determine the level of specific mRNAs; the β-actin gene was used as an internal standard. The specificity of the siRNA action was inspected on the basis of the retention of the mRNA levels of β-actin and its nonhomologous target genes ( *Fig. 1* ).

As can be seen in *Fig. 1* , all siRNAs efficiently and specifically silence their target genes in the cell lines used; maximum silencing (up to 97–99%) being observed 72 h after transfection. siHer decreased the expression of the *Her2 * gene only, having no effect on the expression of the *CCNB1 * and *PKC * genes, as well as that of β-actin. Similar results were obtained when using siRNA siCyc and siPKC. Random-sequence siRNA (siScr) caused no changes in the expression level of target genes, as well.

siHer and siCyc reduced the mRNA levels ofthe *Her2* and *CCNB1 * genes in all of the cell lines used. Seventy-two hours after transfection, the mRNA level of the *Her2 * gene was equal to 15% in KB-3-1 cells, 4% in SK-N-MC cells, and 7% in the MCF-7 cell line with respect to the control. The mRNA level of the *CCNB1 * gene in the КВ-3-1, SK-N-MC, and MCF-7 cells 72 h after transfection with siCyc decreased to 22, 16, and 18%, respectively, as compared to the control. siPKC transfection reduced the mRNA level of the *PKC * gene in MCF-7 cells to 14% ( *Fig. 1* ). The data obtained attest to the fact that siHer, being an inhibitor of the *Her2* gene, has the highest efficiency in SK-N-MC and MCF-7 cell lines, whereas siCyc most considerably decreases the mRNA level of the *CCNB1 * gene in SK-N-MC and MCF-7 cells ( *Fig. 1* ). Starting with day 4 after transfection, the mRNA level of all genes gradually increased and returned to the initial value by day 7–12 after transfection ( *Fig. 1* ).

**Table 1:** The effect of siRNAs on the proliferation  of KB-3-1, SK-N-MC, and MCF-7 cells

siRNA, 200 nM	Proliferation rate*, %		
	KB-3-1	SK-N-MC	MCF-7
Control**	100 ± 7	100 ± 6	100 ± 3
SiScr	113 ± 10	90 ± 7	93 ± 6
SiHer	123 ± 8	36 ± 9	78 ± 2
SiCyc	112 ± 9	14 ± 4	73 ± 2
SiPKC	117 ± 14	9 ± 3	79 ± 2

* The average values over the results of three independent experiments ± the standard deviation are presented.

** Cells treated with a transfection agent only.

As can be seen in *Fig. 1* , the kinetic curves of the relative mRNA level of the *Her2* , *CCNB1* , and  *PKC * genes after transfection with specific siRNAs are U-shaped. The following regions can be visually isolated in each curve: a region corresponding to the decrease in the level of specific mRNA (days 1–3), the region where its amount increases (days 3–7), and the region in which the amount of target mRNA is stabilized at a level corresponding to its level in the control cells (days 7–12). The gradual recovery of the initial mRNA level in cells is probably associated with cell division, which results in the reduction of siRNA concentration in cytoplasm, and, therefore, in attenuation of the RNA interference effect.

Thus, the siRNAs used provide efficient silencing of the *Her2* , *CCNB1* and  *PKC * genes in human tumor cells. The analysis of their expression at the mRNA level has demonstrated the specificity of the action of the siRNAs selected.

**Proliferation of KB-3-1, SK-N-MC, and MCF-7 cell lines after the recovery of the initial level of**

*Her2, CCNB1,* **and** *PKC* **gene expression**

It was shown earlier that the observed anti-proliferative effect of the siRNAs under study is conditioned by the retardation of cell division rather than their death [41]. In this study, we analyzed the changes in the proliferation rate of the cells after the initial level of target gene expression was recovered. The dependence of the proliferation rate of KB-3-1, SK-N-MC, and MCF-7 cells on the expression level of *Her2* , *CCNB1* , and  *PKC * was estimated using the MTT test over a period ranging from day 7 to day 12 after transfection of the corresponding siRNA (200 nm); the cell proliferation rate in the control samples was taken as 100% ( *Table 1* ). As expected, transfection of nonspecific siScr does not result in a reliable change in the cell proliferation rate.

The proliferation rate of the КВ-3-1 cell line after the initial expression levels of target genes ( *Her2* , *CCNB1* ) is virtually the same as that of the control cells. On the contrary, the proliferation rate of SK-N-MC and MCF-7 cells remained low even after the expression of target genes ( *Her2* , *CCNB1* ) was recovered. Thus, after siHer transfection, the division rate of SK-N-MC and MCF-7 cells over the period of 7–12 days was equal to 36 and 78% of its level in the control, respectively. The proliferation rate of SK-N-MC and MCF-7 cells exposed to the action of siCyc remained at a level of 14 and 73% of its level in the control, respectively; that of siPKC was equal to 9 and 79% ( *Table 1* ). It should be mentioned that siPKC has the most pronounced and longest antiproliferative effect (10-fold deceleration of the division rate) on SK-N-MC neuroblastoma cells, in which the expression of the *PKC * gene cannot be detected with the methods used (see [41]). siHer and siCyc had also the most pronounced antiproliferative effect on SK-N-MC line cells. Thus, specific silencing of the genes that are responsible for cell-cycle regulation is capable of considerably decelerating and even terminating SK-N-MC cell division.

As can be seen in *Table 1* , the cell lines used can be conventionally divided into three groups: the cells in which the proliferation rate is completely recovered (КВ-3-1); those in which the proliferation rate remains considerably reduced (SK-N-MC); and those in which the proliferation rate remains insignificantly reduced, after the recovery of the initial mRNA level of the target genes. The temporary silencing of the *Her2* , *CCNB1* , and *PKC * genes is unlikely to result in irreversible changes in the regulation paths of КВ-3-1 cell division; therefore, the cell proliferation rate is recovered, together with the recovery of the mRNA levels of these genes. The situation is different with SK-N-MC cells: the temporary silencing of these genes apparently results in irreversible changes in the paths of proliferation regulation; therefore, the proliferation rate remains considerably reduced even after the mRNA levels of the target genes are recovered. In MCF-7 cells, suppression of proliferation has an intermediate character; the division rate is reduced, although not completely. This fact likely demonstrates that the role of the *Her2* , *CCNB1* , and *PKC * genes in the paths of proliferation regulation differs for these cell lines in terms of its significance.

**Fig. 2:**
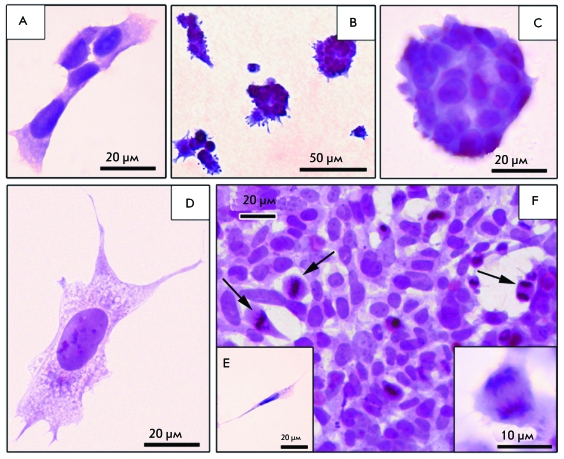
Morphology of neuroblastoma SK-N-MC cells on coverslips (total preparations). A – “islet” cells; B, C – dense spherical cell aggregations; D – neuron-like cell; E – fusiform cell; F – mitoses (indicated by arrows) in the culture cells 5 days after siCyc (200 nM) transfection; the sidebar shows the divergence of the mitotic chromosomes. Staining by hematoxylin.

The results obtained attest to the fact that the *PKC and CCNB1* genes are the most efficient targets for gene-targeted action on SK-N-MC neuroblastoma cells and *CCNB1 * breast cancer cells, respectively. In SK-N-MC cells, the antiproliferative effect of silencing the *CCNB1* and  *PKC * genes is considerably higher than that conditioned by siHer ( *Table 1* ).

**Effect of**

*CCNB1* **and** *PKC* **gene silencing on the morphological characteristics of the SK-N-MC cell culture**

The study demonstrated that the most efficient silencing of the *CCNB1* and  *PKC * genes is observed in a SK-N-MC human neuroblastoma cell culture ( *Table 1* ); therefore, we performed a microscopic study of the changes in the morphology and division of cells of this line under the action of siCyc and siPKC.

A SK-N-MC neuroblastoma cell culture treated with Oligofectamin ^ТМ ^ (control) after 24 h of incubation is represented by different cell types ( *Fig. 2* ). A portion of the cells form a monolayer with “growth islets,” another part of the cells forms small, dense globular aggregates. The “growth islets” of different sizes consist of flat polygonal mononuclear cells with homogeneously stained cytoplasm and non-stained vacuoles ( *Fig. 2A* ) connecting into networks on the coverslip surface. In most cases, the “growth islets” have appreciably distinguishable boundaries; a tendency towards merging being observed. Small spherical cells with a large nucleus, an intensely stained cytoplasm, and smooth surface can be sparsely found in the “growth islets.” Spherical cell aggregates are located on the coverslip both individually and in clusters, their appearance reminding mulberries. Aggregation cells are small, intensely stained, with needle-like or rounded sprouts on the surface; cells with a smooth surface occur, as well ( *Figs. 2B, C* ). The cells lie tightly against each another, making it impossible to count them on total preparations. The third type of cells that are present in the SK-N-MC neuroblastoma cell culture is large spread cells of neuron-like shape with a pale stained vacuolized cytoplasm ( *Fig. 2D). * The cells contain 1–2 nuclei; multinuclear variants occur, as well. Neuron-like cells are mostly localized between the “islet” cells and stand out against the general background by their isolation. Only in extremely rare cases do they contact with the cells of other types, and even if they are localized in the center of the “growth islets,” the space around them is empty.

**Fig. 3:**
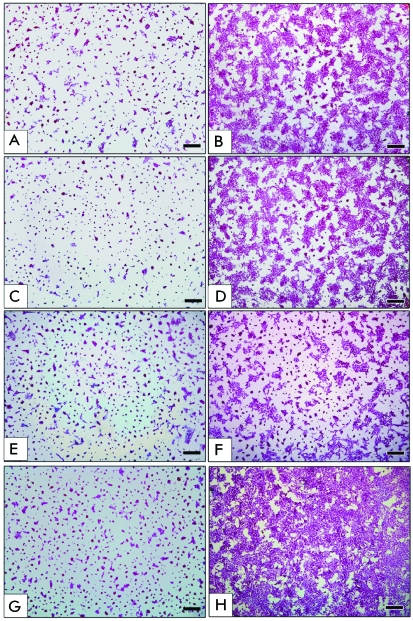
Neuroblastoma SK-N-MC cells on a coverslip (total preparations). Control cells (only transfection reagent treatment): A – 1 day of incubation, B – 3 days of incubation. Transfection with 200 nM siScr: C – 1 day of incubation, D – 3 days of incubation. Transfection with siCyc: E – 3 days of incubation, F – 12 days of incubation. Transfection with siPKC: G – 3 days of incubation, H – 12 days of incubation. Staining with hematoxylin. Size bar is 200 μm.

Small, strongly elongated spindle- or needle-shaped cells with sparse vacuoles in the cytoplasm also occur in the SK-N-MC neuroblastoma cell culture ( *Fig. 2E* ). Thus, four morphological types of cells are revealed in preparations of SK-N-MC neuroblastoma cells at the light-optical level, no transition forms being observed upon the used method of analysis.

When incubating SK-N-MC cells treated with Oligofectamin ^ТМ^ for 24–72 h, the morphological characteristics of the cell types remain constant; all of the described variants being revealed on the coverslips. A considerable increase in the total amount of cells on coverslips is observed due to the “islet” cells; the “growth islets” merge, and by the end of day 3 of incubation these cells represent the main mass of the culture. Multiple mitoses are observed in the “islet” SK-N-MC culture cells during the incubation for 24–72 h ( *Table 2* ). The variety of sizes of spherical cell aggregates, neuron-like and spindle-like cells does not noticeably change.

The comparison of preparations of intact SK-N-MC cells and Oligofectamin ^ТМ^ -treated cells revealed no observable changes in the morphological characteristics of the cells. Treatment with a transfection agent did not result in the emergence of new variants of cells or a noticeable change in their ratio. Transfection of SK-N-MC cells with all siRNAs did not result in the emergence of new morphological cell types, either: all preparations contained the cell varieties described above at all incubation periods. Transfection of SK-N-MC cells with siScr did not result in any noticeable changes in the ratio between different types of cells during 72 h of incubation, as compared with preparations of the oligofectamin-treated culture.

A study of the preparations of SK-N-MC neuroblastoma cells obtained after different time intervals after transfection revealed distinct morphological signs of the effect of siCyc and siPKC on the vital activity of the cells. The “seeding” dosage being equal, the cells either Oligofectamin ^ТМ^ -treated or transfected with siScr almost completely filled the surface area of the coverslip after 3 days of incubation ( *Figs. 3B,D* ), whereas transfection with siCyc or siPKC abruptly decelerated cell division, their number on the coverslip on the same day being incomparably smaller ( *Figs. 3E,G* ). The cells transfected with siCyc and siPKC were incubated for 12 days ( *Figs. 3F,H* ), whereas it was necessary to reseed the cells transfected with siScr and the control cells once in 3 days. The differences in the growth rate and necessity to reseed the control and siScr transfected cells rendered it impossible to compare the morphological characteristics of the culture transfected with siCyc and siPKC with the control preparations after 5–12 days of incubation. Only the preparations incubated for 72 h after transfection could be compared. The preparations of SK-N-MC cells transfected with siCyc and siPKC during the period contained an incomparably smaller number of “islet” cells, in comparison to the control. The major part of the population was represented by small, intensely stained cells from the globular aggregates, which included several cells at a time. Against the background of a decreasing number of “islet” cells, the relative number of neuron-like and fusiform cells was increasing. Similar to events in the control preparation, mitoses were revealed mostly in the “islet” cells; their number abruptly decreasing, especially if compared with the preparations transfected with siScr ( *Table 2* ).

**Table 2:** The number of mitoses in SK-N-MC cells 1–12 days after transfection with siCyc and siPKC (200 nm)

Day	Number of mitoses per 1,000 cells				
	Control*	SiScr	SiCyc	SiPKC	Control without transfecting agent
1	14 ± 5	16 ± 8	-	-	-
2	40 ± 16	25 ± 7	-	-	22 ± 15
3	21 ± 10	33 ± 12	15 ± 11	16 ± 7	-
5	-	-	30 ± 12	20 ± 11	-
7	-	-	28 ± 6	35 ± 5	-
12	-	-	30 ± 6	49 ± 14	-

* Cells treated with a transfection agent only.

(-) – not determined.

Thus, 72 h after transfection with siCyc and siPKC, the mitotic activity of “islet” cells is considerably decreased in neuroblastoma cells and their number decreases; therefore, the ratio between the morphological types of culture cells changes and the share of neuron-like and fusiform cells increases.

Late effects (days 5–12) of the transfection of neuroblastoma cells with siCyc and siPKC differed to a certain extent. When using siCyc, the number of mitoses increased 5 days after transfection and remained at the same level over the entire observation period ( *Table 2* ). The cells at the mitosis stage were mostly revealed among “islet” cells ( *Fig. 2E* ), their number increasing, while the number of spherical cell aggregates, neuron-like and fusiform cells had not noticeably changed. It should be noted that even after 12 days of incubation, the number of neuroblastoma cells on the coverslips and the number of “islet” cells were appreciably smaller than that in the control 72 h after the transfection ( *Fig. 3B,D,F* ). Transfection of neuroblastoma cells with siPKC resulted in a more prolonged decrease in the mitosis number; its growth being recorded only 7 days after transfection. It continued until the end of observations ( *Table 2* ). Similar to the case of transfection with siCyc, mitoses were revealed mostly in the islet cells; their number increasing during the incubation. Five days after transfection with siPKC, the relative increase in the number of neuron-like and fusiform cells noted after 3 days remained constant; the number of spherical cell aggregations also did not change. The islet cells comprised the major part of SK-N-MC neuroblastoma cells 7 and 12 days after transfection; their number increased intensely due to the active mitotic division ( *Table 2* ). The density of cells on the coverslip 12 days after the transfection was at a maximum and exceeded that in the control and upon transfection with siCyc ( *Fig. 3B,D,F,H)* . Thus, transfection with siPKC results in a more prolonged blocking of mitotic division of SK-N-MC neuroblastoma cells, in comparison with siCyc transfection; however, further growth in the number of mitoses is more pronounced and results in a more intense cell culture growth. The analysis of the morphological characteristics of SK-N-MC neuroblastoma demonstrates that “islet cells” comprising the main part of the cell population, their division being suppressed, are the main target of siCyc and siРКС. The effect of siPKC appears to be more complex as compared with that of siCyc, requiring further investigation. The results of a microscopic study of SK-N-MC cell growth after silencing of the *CCNB1* and  *PKC * genes ( *Fig. 3* ) agree well with the data on the effect of this silencing on the proliferation rate of these cells ( *Table 1* ).

## DISCUSSION

Today, interfering RNAs are regarded as potential therapeutic agents; a number of preparations based on them have been going through different stages of clinical trials [48]. An urgent problem is revealing the targets in which the short-term silencing results in irreversible consequences for a cancer cell, such as terminal differentiation, apoptosis, or long-term suppression of their proliferation. An appreciably large body of data on successful temporal silencing of the genes responsible for the emergence of oncologic diseases, including the *Her2* , *CCNB1,* and  *PKC* genes, has been published recently [4, 6, 7, 49]. It has been demonstrated that this silencing results in a decrease in the proliferation rate of cancer cells; however, most experiments were confined to incubation of up to 96 h, whereas the fall in the mRNA level of the target gene under the action of siRNA is retained for up to 4–5 days. There had been no data concerning the duration of the effect of siRNA on *Her2, CCNB1, * and *PKC* gene expression; therefore, we were the first to study the changes in the expression level of these genes 12 days after transfection with specific siRNAs. We were able to demonstrate that a maximum decrease in the expression level of the *Her2* , *CCNB1* , and *PKC * genes in KB-3-1, SK-N-MC, and MCF-7 cells is observed 72 h after siRNA transfection. The silencing of these genes, as it was earlier shown in [41], slows to a different extent the proliferation of tumor cells from day 3 to day 7 of their cultivation after siRNA transfection. The analysis of the kinetics of the changes in the levels of specific mRNAs has demonstrated that the initial mRNA levels are recovered as early as on day 5–day 7 after a single transfection with the corresponding siRNA, which is likely determined by the lifetime of siRNA in a cell ( *Fig. 1* ). The duration of the antiproliferative effect of siRNA in various cell lines considerably varies. The determination of the cell proliferation rate over a period of day 7–day 12 after siRNA transfection demonstrated that after the initial level of the target gene expression is recovered, the proliferation rate of КВ-3-1 cells does not differ from the rate of cell growth in the control (that of nontreated cells, that of the cells subjected to the action of the transfection agent only, and those transfected with a random siRNA (SiScr)). The growth of MCF-7 cells transfected with specific siRNAs remained low to a certain extent as compared with the control. Thus, regardless of the pronounced antiproliferative effect of siRNA preparations revealed, in order to limit the growth of these cell lines, it is necessary to sustain the decreased level of target gene expression. This can be achieved by additional introduction of the corresponding siRNA into the cells or using short interfering hairpin RNA (shRNAs), which are expressed immediately in the target cells after their transduction with recombinant viruses (adeno-, adeno-associated , lenti- and retroviruses). However, the issue of safety in using recombinant viruses for therapeutic purposes remains far from settled [50–52].

We found that the temporary silencing of the *Her2, CCNB1, * and *PKC * genes in a SK-N-MC cell culture results in considerable slowing of cell division even after the initial mRNA level of target genes is recovered ( *Table 1* ). The *CCNB1 * and  *PKC * genes serve as the most efficient target to achieve long-term suppression of proliferation in SK-N-MC neuroblastoma cells ( *Table 1* ). The pronounced antiproliferative effect (5–10 times) of the short-term silencing of these genes remains for up to 12 days of incubation. It should be mentioned that the long-term antiproliferative effect of siPKC on SK-N-MC cells is quite unexpected, since no hyperexpression of the *PKC * gene is observed in these cells [41]. Nevertheless, the long-term antiproliferative effect of this specific inhibitor in SK-N-MC cells has been demonstrated; the elucidation of its reasons requires additional studies.

The proliferation rate of SK-N-MC cells remains at a considerably low level even after siCyc and siPKC have been removed from the cells and the level of their target genes returns to the initial level. This result is rather unexpected. It appears that this phenomenon can be interpreted taking into account the specific pattern of gene expression in cells of neuronal origin (to which SK-N-MC cells belong), the changes in the expression pattern under the action of the temporary silencing of target genes, and its subsequent reactivation. Thereby, even a short-term expression inhibition of the *CCNB1 * or * PKC * gene is likely to result in a very slow recovery of the cell proliferation rate (or in its complete blocking) even after the initial level of the products of each target gene is recovered. It is already known that a deficiency in protein p53 is observed in this cell line; i.e., they do not contain the most important participant of the apoptosis induction chain, which appears to play a significant role in their uncontrollable proliferation [53]. It is possible that the absence of this participant in the regulatory cascade is one of the key reasons behind the difference between the consequences of a short-term inhibition of *CCNB1 * and * PKC * gene expression in this cell line and the other ones that were used; their proliferation rate having recovered after the level of the target gene had returned to its initial level. It would be of interest to study the joint action of siRNAs and p53 inhibitors on the proliferation of cells of different lines. As shown by the results obtained, after the inhibition of *CCNB1 * and * PKC * gene expression in a SK-N-MC cell culture, the share of neuron-like and fusiform cells characterized by a lower mitotic activity as compared with other morphological cell types in the culture was high to a certain extent. An increase in the share of these cells may attest to the induction of the initial events of cell differentiation (either reversible or irreversible), which may also be one of the reasons for the so prolonged antiproliferative effect of the selected siRNAs. To verify these hypotheses, it is necessary to study the behavior of the cells as exposed to the action of siRNAs for a longer period of time. Nevertheless, the data on long-term proliferation suppression may point to an important role played by the *CCNB1 * and *PKC * genes in the aggressive proliferation of SK-N-MC.

A study of the morphology of SK-N-MC cells 3–12 days after transfection has demonstrated that temporary inhibition of the expression of the *Her2, CCNB1,* and * PKC * genes does not result in their death or terminal differentiation (which is demonstrated by the retention of different cell types in the population) and slows cell division. The gradual increase in the total amount of cells in the preparations transfected with specific siRNAs and, in particular cells at the mitosis stage by days 10–12 of incubation, points to the fact that the duration of the antiproliferative effect of these siRNAs on neuroblastoma cells is likely limited to 12–15 days.

In this study, it was demonstrated using genetic and morphological material that the *Her2* , *CCNB1* , and  *PKC* genes are efficient targets for specifically addressed siRNAs in neuroblastoma cells, since the decrease in the expression level of target genes attained with their aid results in efficient and long-term proliferation inhibition. The use of siRNAs to control the growth of tumor cells that survive chemotherapy may become one of the aspects of a complex therapy upon cancer and neuroblastoma, in particular. Until recently, low-molecular-weight compounds found empirically have been used as antitumor agents. Interfering RNAs have the potential to become the new generation of preparations that considerably outperform known ones in terms of specificity, efficacy, and nontoxicity. 

## Abbreviations

C: 2’-O-methylcytosine
U: 2’-O-methyluridine
siRNA: small interfering RNA
МТТ: 3-[4,5-dimethylthiazol-2-yl]-2,5-diphenyltetrazolium bromide
PBS: phosphate buffered saline
FBS: fetal bovine serum
